# The Effect of College Students' Adaptability on Nomophobia: Based on Lasso Regression

**DOI:** 10.3389/fpsyt.2021.641417

**Published:** 2021-10-29

**Authors:** Jing Luo, Shixiu Ren, Yuxin Li, Tour Liu

**Affiliations:** ^1^Department of Mathematics and Statistics, South Central University for Nationalities, Wuhan, China; ^2^Collaborative Innovation Center of Assessment for Basic Education Quality, Beijing Normal University, Beijing, China; ^3^Key Research Base of Humanities and Social Sciences of the Ministry of Education, Academy of Psychology and Behavior, Tianjin Normal University, Tianjin, China; ^4^Faculty of Psychology, Tianjin Normal University, Tianjin, China; ^5^Tianjin Social Science Laboratory of Students' Mental Development and Learning, Tianjin, China

**Keywords:** mobile phone addiction, nomophobia, adaptability, machine learning, Lasso regression

## Abstract

Smartphones can improve our lives, but also consume our lives. It is known that problematic mobile phone use, such as nomophobia, can lead to some mental health problems. So far, psychological factors behind nomophobia were yet to be fully discovered. Previous studies showed that individuals' adaptability was closely related to nomophobia. However, adaptability was a complex construct that contains various components, and it was unclear whether these components contributed equally to nomophobia. This study investigated 678 college students by using Chinese versions of the nomophobia questionnaire, mobile phone addiction tendency scale, and freshmen adaptability scale. Lasso regression was used to further explore the key factors that could affect nomophobia. Model results showed that the value of λ+1se was [0.303, 0.423] at the minimum mean squared error in the training data. Emotional adaptability significantly predicted the fear of being unable to access information (β = −0.022, *p* < 0.001), losing convenience (β = −0.067, *p* < 0.001), and losing Internet connection (β = −0.003, *p* < 0.01) after λ+1se was included in the testing data, and the *R*^2^ were 0.496, 0.483, and 0.493. Homesickness adaptability significantly predicted the fear of losing contact (β = −0.056, *p* < 0.05), and *R*^2^ was 0.508. In addition, similar results were obtained by using datasets of mobile phone addiction and adaptability. Therefore, we concluded that the emotional adaptability has an important effect on nomophobia. Additionally, we also found that homesickness adaptability has an important role in predicting fear of losing contact.

## Introduction

Adaptability is an important psychological trait for college students (1). It refers to a soft skill that can help people to rapidly learn skills and behaviors in response to changing circumstances (2). Regina et al. (3) found that adolescents' adaptability facilitates positive wellbeing when they need to adapt to a rapidly changing environment.

Quintas-Hijos et al. (4) examined college students' adaptability and its consequences since it significantly influences the development of college students. Yang et al. (5) also found that there was a significant association between adaptability and coping style. Xie et al. (6) also showed similar findings that students' adaptability in school could significantly predict their academic performance. Chen et al. (7) discovered that adaptability could significantly influence college students' life satisfaction and mental health symptoms, such as anxiety and depression (8). Altogether, adaptability is a possible contributing factor that affects development of early adulthood (9, 10).

Researchers have extensively studied the associations between adaptability and mobile phone use and they found that there was a close link between them (11). Meghan et al. (12) found that adaptability was an important predictor for social media addiction by using a traditional regression model. Other studies also supported this conclusion by using different datasets in different studies (13, 14).

There were many studies about mobile phone addiction (15), but no universally acknowledged criterions or symptoms for mobile phone addiction in DSM-V or ICD-11 (16, 17). Gradually, the concept of mobile phone addiction was discarded (18) and replaced by problematic mobile phone use (PMPU) (19). Recently, nomophobia, a newer PMPU-related notion, has been proposed based on the Fear of Missing Out Theory (FOMO) (20–22). It was defined as distress or anxiety when an individual loses access to their mobile phone, such as from battery drain or inability to use while in class (23, 24). It has been widely studied as a by-product of emerging technologies (25).

There were many studies about adaptability and mobile phone addiction (11), but only one study was about adaptability and nomophobia. A study of Bragazzi et al. (26) discovered that individual's maladaptive coping style could significantly predict nomophobia. Theoretically, the relationship between nomophobia and adaptability could be explained by the Interaction of Person-Affect-Cognition-Execution (I-PACE) model.

The Interaction of Person-Affect-Cognition-Execution (I-PACE) Theory (27) described the psychological and neurobiological processes underlying the development of mobile phone overuse, such as gaming, gambling, viewing pornography, buying shopping, and social-networking addictions. A great number of studies had supported I-PACE in modeling mobile phone overuse (28). I-PACE proposed that many factors could affect mobile phone overuse (e.g., mobile phone addiction and nomophobia), including biological factors (e.g., gender), personality factors, and psychological factors (e.g., adaptability). The present study, therefore, was carried out to examine the extent to which adaptability affected nomophobia by establishing the I-PACE model.

In general, many investigators have used ordinary least square (OLS) to estimate the relationship among variables in the regression model (29). The proposed method provides the most accurate and unbiased estimation by the sum of the minimum residuals (30), but it also has some shortcomings, such as overfitting results and poor predictions on future observations (31). In fact, those problems are even more serious when there are many predictors in a regression model (29, 32, 33).

Regularization methods, such as ridge regression and Lasso regression, in machine learning have been used to make up for the limitations of OLS. Algorithms in machine learning are divided into supervised and unsupervised algorithms (34). In unsupervised learning, the machine uses unlabeled data and learns by itself without any supervision (35). It can be further grouped into clustering and association (36). In supervised learning, the machine learns under supervision, and it has training data and testing data (37). The computer learns and chooses an optimal model by using training data and then gets the final results by fitting the optimal model with testing data. Supervised learning is divided into classification and regression (34). Lasso regression (32, 38) used in this study is a kind of regression that used least angle regression algorithm instead of least squares.

Least absolute shrinkage and selection operator (Lasso) (38) is one of the best regularization methods to deal with overfitting problems and get more accurate results (39). The proposed method can shrink the small coefficient toward zero by adding a penalty term in the process of model estimation (40). Consequently, it can obtain higher model prediction accuracy and model generalizability (32). Moreover, Lasso regression can help researchers to perform variable selection and help them to get more concise and more efficient models (32, 41). This method plays an important role in the construction and perfection of psychological theory. The Lasso formula can be described as follows.


$$\begin{array}{l} \overset{n}{\underset{i=1}{\sum }}{\left(y_{i}-\beta_{0}-\overset{p}{\underset{j=1}{\sum }}\beta_{j}x_{ij}\right)}^{2}+\lambda \overset{p}{\underset{j=1}{\sum }}|\beta_{j}|=RSS+\lambda \overset{p}{\underset{j=1}{\sum }}|\beta_{j}| \end{array}$$

where *i* = 1, …, *n* denotes the number of the observations; *j* = 1, …, *p* denotes the number of the predictors; β_0_ denotes the intercept in the linear regression model; β_*j*_ denotes the regression coefficient about the *j*th predictors and response; λ denotes the penalty parameter.

### Present Study

The main purpose of this study was to explore the effect of adaptability on nomophobia. Moreover, both adaptability and nomophobia were multidimensional. Cao and Mao (2) proposed a six-dimension construct for adaptability, and Ren et al. (42) proposed that nomophobia contained four dimensions. How different dimensions of adaptability could affect the different nomophobia facets was a main issue to discuss in this study. We believed that individuals' emotional cognition could affect their decisions and behaviors, and then resulted in some problematic mobile phone use behaviors according to the I-PACE (43). Therefore, we assumed that individuals' gender and adaptability could affect nomophobia.

Researchers usually used ordinary least square to explore the relationships between variables in previous studies. It led to model-data overfit and multi-collinearity when many predictors were included in models. Therefore, the Lasso regularization method in machine learning was conducted in our empirical study to explore the key predictors that affect nomophobia.

### Aims

Our primary aim was to explore the key factors (dimensions) of adaptability that could predict nomophobia by using the Lasso regularization method based on a sample of Chinese college students. Then, we re-examined the role of those key dimensions in the relationship between mobile phone addiction and adaptability. We hypothesized that there were significant associations between nomophobia and adaptability, and emotional adaptability and homesickness adaptability could significantly predict nomophobia. The current study examined the extent to which individuals experience nomophobia and sees whether our finding is generalizable.

## Methods

### Participants and Procedure

We recruited 678 volunteers to complete a 5–8-min survey through online and paper-and-pencil questionnaires in 2019 before the outbreak of COVID-19. Both online and offline surveys were used in this study. Fifty participants completed their questionnaires as a paper-and-pencil version in a classroom. The other participants completed online surveys through the Wen Juan Xing App (https://www.wjx.cn). Before data analysis, measurement invariance was supported between the paper-and-pencil dataset and the online dataset by a multi-group confirmatory factor analysis. Consequently, those two datasets had been analyzed together.

Before we analyzed our data, five participants (0.74%) were removed because of missing more than 20 items. Missing values were imputed by expectation maximization (EM) method due to the fact that missing values were missing completely at random (MCAR). A total of 673 participants were included in the data analysis (20.5% men, 79.5% women; *M* = 20.4, *SD* = 1.3). One hundred sixty students were freshmen, as well as 196 sophomores, 210 juniors, 71 seniors, and 36 post-baccalaureates. The study involving human participants was reviewed and approved by the ethics committee of Tianjin Normal University in China (Ethical review number: XL2020-08). The participants provided their written informed consent to participate in this study.

### Measures

#### Instruments

##### Chinese Version of the Nomophobia Questionnaire

Based on the original nomophobia questionnaire of Ren et al. (42) and Yildirim and Correia (44) revised the Chinese version of the nomophobia questionnaire (NMP-C). In that study, exploratory structural equation modeling (ESEM) and item response model (IRT) were used to perform item selection and to explore the structure of nomophobia scale, and confirmatory factor analysis (CFA) was conducted to verify this structure. The NMP-C contains 16 items and four dimensions, including fear of being unable to access information, losing convenience, losing contact, and losing Internet connection. It used a seven-point Likert scale, ranging from 1 (“Not meet at all”) to 7 (“Completely in conformity with”) (42). Cronbach's α for the whole scale was 0.931 and that for the four dimensions ranged from 0.789 to 0.901; the ω of the whole scale was 0.931 in this study.

##### Mobile Phone Addiction Tendency Scale

We wanted to re-examine the role of those key dimensions in the relationship between mobile phone addiction and adaptability. So, we also used Mobile Phone Addiction Tendency Scale (MPATS) to improve the accuracy and reliability of results. MPATS was developed by Xiong et al. (45). The MPATS is composed of 16 items and four factors, including withdrawal symptoms, salience, social comfort, and mood changes. It was a five-point Likert scale, ranging from 1 (“Extremely inconsistent”) to 5 (“Extremely consistent”). Jang and Bai (46) found that the Cronbach's α of scale was 0.830 and that for the four dimensions ranged from 0.810 to 0.920. Cronbach's α for the whole scale was 0.896 and that for the four dimensions were from 0.615 to 0.803 in this study. The ω of the whole scale was 0.897 in this study.

##### The Freshmen Adaptability Scale

The freshmen adaptability scale was originally developed for freshmen by Cao and Mao (2), and it was revised by Luo (47). The scale was shown to be both valid and reliable across settings [e.g., (48)]. The scale has 24 items, including learning, professional, homesickness, interpersonal, emotional, and economical adaptability. It was a six-point Likert scale, ranging from 1 (“Extremely inconsistent”) to 6 (“Extremely consistent”). Cronbach's α for the whole scale was 0.815 and that for the four dimensions ranged from 0.720 to 0.837 in this study; the ω of the whole scale was 0.771.

#### Software and Statistical Methods

SPSS26.0 was used for data preprocessing and internal consistency analysis. Traditional multiple linear regression and sparse linear regression model was conducted in R-4.0.5 (49). OLS was used in traditional regression model, and the Lasso shrinkage algorithm in Machine Learning was used in sparse linear regression model. At first, the sample was divided into two halves by using function of sample randomly, one-half as a training dataset (*n* = 336) and the other as a testing dataset (*n* = 337). Next, training data were conducted to get the best λ by using the cross-validation approach, and then the best λ was taken into the testing data to get the final Lasso regression by using *glmnet* packages. Finally, the *covTest* package was used to test the significance of coefficients in Lasso regression.

## Results

### Correlations Between Nomophobia and Adaptability

Table 1 shows the descriptive statistics of nomophobia and adaptability among our participants. Results showed that fear of being unable to access information showed a mean (*M*) of 4.162, *SD* = 1.272 (maximum of seven), fear of losing convenience showed *M* = 4.215, *SD* = 1.460, fear of losing contact showed *M* = 4.221, *SD* = 1.488, and fear of losing Internet connection showed *M* = 3.759, *SD* = 1.502. Besides that, learning adaptability showed *M* = 3.782, *SD* = 0.923 (maximum of six), professional adaptability showed *M* = 4.014, *SD* = 1.097, homesickness adaptability showed *M* = 3.232, *SD* = 1.117, interpersonal adaptability showed *M* = 4.413, *SD* = 0.895, emotional adaptability showed *M* = 3.987, *SD* = 1.005, and economical adaptability showed *M* = 4.157, *SD* = 1.020.

**Table 1:** Descriptive statistics for the nomophobia and adaptability.

**Variable**	**Total sample (*****n*** **= 673)**	
	***M***	***SD***
Unable to access information	4.162	1.272
Losing convenience	4.215	1.460
Losing contact	4.221	1.488
Losing Internet connection	3.759	1.502
Learning adaptability	3.782	0.923
Professional adaptability	4.014	1.097
Homesickness adaptability	3.232	1.117
Interpersonal adaptability	4.413	0.895
Emotional adaptability	3.987	1.005
Economical adaptability	4.157	1.020

**Figure 1:**
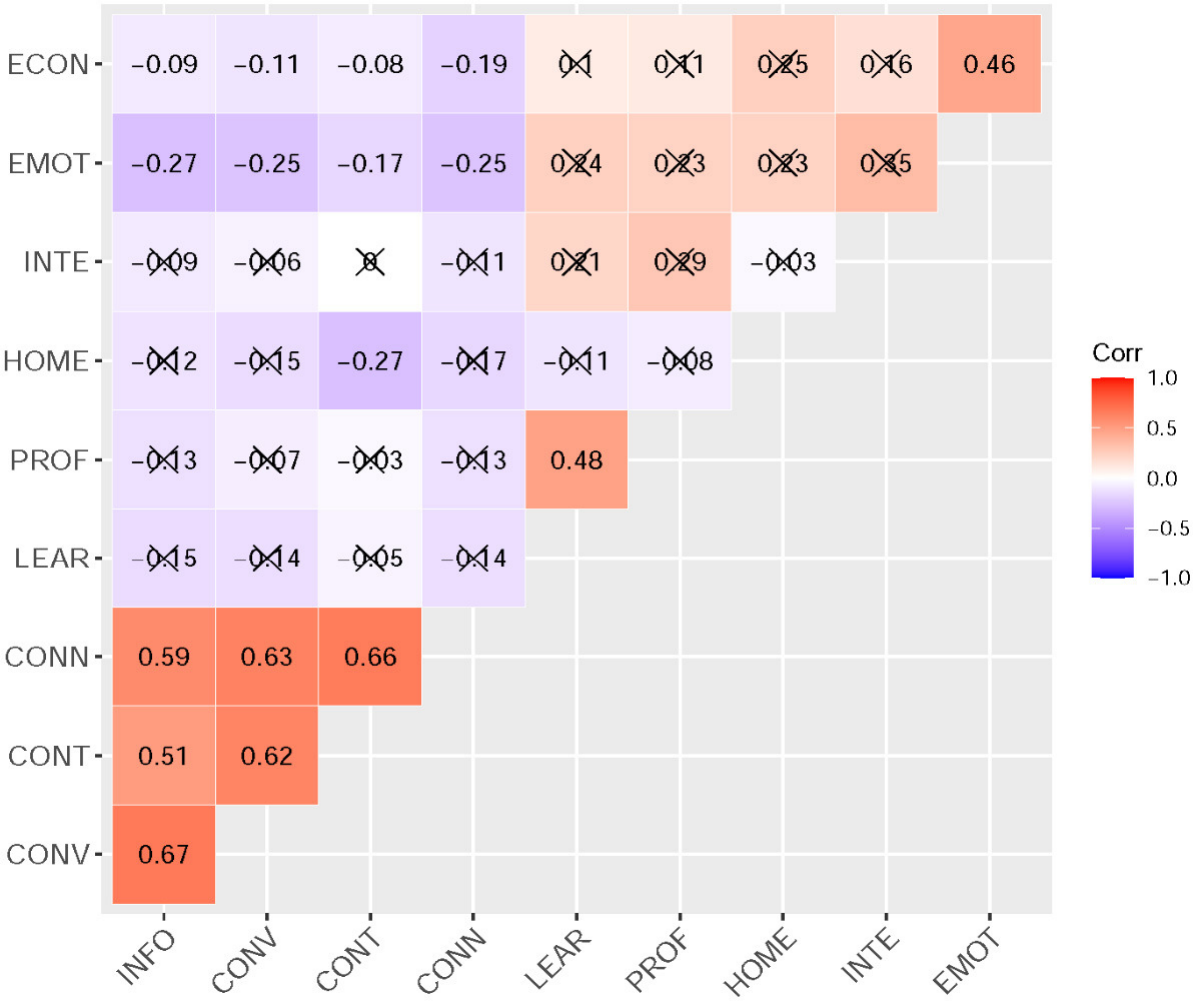
Correlations between nomophobia with adaptability. INFO, unable to access information; CONV, losing convenience; CONT, losing contact; CONN, losing Internet connection; LEAR, learning adaptability; PROF, professional adaptability; HOME, homesickness adaptability; INTE, interpersonal adaptability; EMOT, emotional adaptability; ECON, economical adaptability.

Pearson correlation matrix was conducted to analyze the correlations between variables. Bivariate correlations for summed scores were reported in Figure 1. There were significant correlations between homesickness adaptability and fear of being unable to access information (*r* = −0.123, *p* < 0.01), losing convenience (*r* = −0.152, *p* < 0.001), losing contact (*r* = −0.274, *p* < 0.001), and losing Internet connection (*r* = −0.168, *p* < 0.001). Results also showed that emotional adaptability negatively associated with being unable to access information (*r* = −0.267, *p* < 0.001), losing convenience (*r* = −0.253, *p* < 0.001), losing contact (*r* = −0.172, *p* < 0.001), and losing Internet connection (*r* = −0.254, *p* < 0.001). Besides that, learning adaptability and economical adaptability were negatively related to fear of being unable to access information (*r* = −0.146, *p* < 0.001; *r* = −0.094, *p* < 0.05), losing convenience (*r* = −0.136, *p* < 0.001; *r* = −0.111, *p* < 0.01), and losing Internet connection (*r* = −0.140, *p* < 0.001; *r* = −0.190, *p* < 0.001). Meanwhile, professional adaptability and interpersonal adaptability were related to the level of fear of being unable to access information (*r* = −0.132, *p* < 0.01; *r* = −0.191, *p* < 0.05) and losing Internet connection (*r* = −0.130, *p* < 0.01; *r* = −0.110 *p* < 0.01). Other concrete results can be seen in Figure 1.

### Gender Difference in Nomophobia and Adaptability

*t*-test was conducted to evaluate the association between gender and our study variables. Results showed that gender was significantly associated with fear of being unable to access information *t*_(671)_ = −2.655, *p* < 0.01 (Men: *M* = 3.906, *SD* = 1.223; Women: *M* = 4.228, *SD* = 1.278), losing convenience *t*_(671)_ = −3.288, *p* < 0.01 (Men: *M* = 3.853, *SD* = 1.353; Women: *M* = 4.308, *SD* = 1.474), and losing contact *t*_(671)_ = −3.521, *p* < 0.001 (Men: *M* = 3.827, *SD* = 1.449; Women: *M* = 4.323, *SD* = 1.483). However, there was no difference in the level of fear of losing Internet connection *t*_(671)_ = −1.142, *p* = 0.158 (Men: *M* = 3.598, *SD* = 1.478; Women: *M* = 3.800, *SD* = 1.507).

In addition, we also found that there was a significant association between gender and homesickness adaptability *t*_(671)_ = 5.047, *p* < 0.001 (Men: *M* = 3.652, *SD* = 1.070; Women: *M* = 3.124, *SD* = 1.104) and economical adaptability *t*_(671)_ = −2.480, *p* < 0.05 (Men: *M* = 3.965, *SD* = 1.043; Women: *M* = 4.206, *SD* = 1.010).

### Traditional Multiple Regression Model of Adaptability on Nomophobia

The collinearity diagnostics was used to test multicollinearity among the independent variables before the traditional multiple regression. The VIF values [1.079, 1.519] supported that there was no multicollinearity. Traditional regression, which is based on least squares algorithm, was chosen to explore the factors that could predict nomophobia. Gender was dummy coded before the regression analysis. Gender and six dimensions of adaptability were taken as predictors, and four dimensions of nomophobia were response variables. Table 2 shows four traditional regression models.

**Table 2:** Traditional multiple regression model about nomophobia and adaptability.

**Predicted variables**	**Response variables**			
	**Access information**	**Losing convenience**	**Losing contact**	**Losing Internet connection**
Intercept	5.579^***^	5.535^***^	5.290^***^	6.349^***^
Gender	0.226	0.341^*^	0.294^*^	0.111
Learning adaptability	−0.100	−0.159^*^	−0.065	−0.126
Professional adaptability	−0.073	−0.001	−0.033	−0.078
Homesickness adaptability	−0.086	−0.123^*^	−0.322^***^	−0.162^**^
Interpersonal adaptability	0.029	0.067	0.085	−0.022
Emotional adaptability	−0.229^***^	−0.316^***^	−0.190^**^	−0.228^***^
Economical adaptability	0.045	0.008	0.050	−0.113

*** *p < 0.001*,

** *p < 0.01, and*

* *p < 0.05*.

Results about four traditional regression models in Table 2 showed that emotional adaptability was negatively associated with four dimensions of nomophobia (βϵ[−0.190, −0.316], *p* < 0.01). Homesickness adaptability was negatively associated with the fear of losing convenience (β = −0.123, *p* < 0.05), fear of losing contact (β = −0.322, *p* < 0.001), and fear of losing Internet connection (β = −0.162, *p* < 0.01). In addition, learning adaptability was negatively associated with the fear of losing convenience (β = −0.159 *p* < 0.05). Gender was positively associated with the fear of losing convenience (β = 0.341, *p* < 0.05) and losing contact (β = 0.294, *p* < 0.05).

### Lasso Regression Model of Adaptability on Nomophobia

Traditional multiple regression results showed that the nomophobia was related to the learning adaptability, homesickness adaptability, emotional adaptability, and gender. Therefore, Lasso regression based on least angle regression algorithm was used to confirm the key factors that could predict the nomophobia by using R package *glmnet*.

Gender and six dimensions of adaptability were taken as predictors, and four dimensions of nomophobia were response variables. Therefore, there were four Lasso regression models for this section, and they are reported in Table 3. The values of λ were 0.001, 0.020, and 0.045 and λ+1se values were 0.338, 0.303, and 0.423 at the minimum mean squared error (MSE) through the training dataset when response data were from fear of being unable to access information, losing convenience, and losing Internet connection. λ+1se was considered to be the best λ because the penalty power of λ was too small to solve the problem of overfitting. Finally, results showed that emotional adaptability could predict fear of being unable to access information (β = −0.022, *p* < 0.001), fear of losing convenience (β = −0.067, *p* < 0.001), and fear of losing Internet connection (β = −0.003, *p* < 0.01), and it was accepted by *covTest* package; *R*^2^ values were 0.496, 0.483, and 0.493, and *MSE* values were 1.391, 2.046, and 2.184. λ+1se was 0.345 when responses were from fear of losing contact, and the retained variable was homesickness adaptability (β = −0.056, *p* < 0.05); *R*^2^ was 0.508, *MSE* was 2.032, and the results can be seen in Table 3.

**Table 3:** Lasso regression model of nomophobia and adaptability.

**Predicted variables**	**Response variables**			
	**Access information**	**Losing convenience**	**Losing contact**	**Losing Internet connection**
Intercept	4.252^***^	4.481^***^	4.401^***^	3.771^***^
Gender	–	–	–	–
Learning adaptability	–	–	–	–
Professional adaptability	–	–	–	–
Homesickness adaptability	–	–	−0.056^*^	–
Interpersonal adaptability	–	–	–	–
Emotional adaptability	−0.022^***^	−0.067^***^	–	−0.003^**^
Economical adaptability	–	–	–	–

*** *p < 0.001*,

** *p < 0.01, and*

* *p < 0.05*.

### The Lasso Regression Model of Adaptability on Mobile Phone Addiction

The dataset of mobile phone addiction and adaptability was used to verify the role of emotional adaptability and homesickness adaptability. In this part, collinearity diagnostics was used to test multicollinearity. Results showed that there was no multicollinearity among the independent variables in this study (VIFϵ[1.079, 1.519]).

Firstly, we performed the traditional regression model about adaptability and mobile phone addiction. Therefore, gender and six dimensions of adaptability were taken as predictors, and four dimensions of mobile phone addiction were response variables. Gender was dummy coded before the regression analysis.

Multiple regression results in Table 4 showed that emotional adaptability was negatively associated with the level of withdrawal symptoms (β = −0.193, *p* < 0.001), salience (β = −0.148, *p* < 0.001), social comfort (β = −0.321, *p* < 0.01), and mood changes (β = −0.153, *p* < 0.001). Homesickness adaptability and learning adaptability were negatively associated with the withdrawal symptoms (βϵ[−0.142, −0.073], *p* < 0.05), salience (βϵ[−0.225, −0.149], *p* < 0.001), and mood changes (βϵ[−0.129, −0.111], *p* < 0.01).

**Table 4:** Traditional multiple regression and Lasso regression model of mobile phone addiction and adaptability.

**Predicted variables**	**Traditional multiple regression**				**Lasso regression**			
	**Withdrawal symptoms**	**Salience**	**Social comfort**	**Mood changes**	**Withdrawal symptoms**	**Salience**	**Social comfort**	**Mood changes**
Intercept	4.478^***^	5.330^***^	5.064^***^	5.147^***^	3.326^***^	4.089^***^	3.372^***^	3.336^***^
Gender	−0.001	0.104	−0.041	0.062				
Learning adaptability	−0.073^*^	−0.225^***^	−0.078	−0.111^**^		−0.159^***^		−0.007
Professional adaptability	−0.068^*^	−0.055	−0.012	−0.042		−0.013		
Homesickness adaptability	−0.142^***^	−0.149^***^	−0.048	−0.129^***^	−0.005^**^	−0.065^**^		−0.008
Interpersonal adaptability	−0.005	−0.037	−0.035	−0.081^*^				
Emotional adaptability	−0.193^***^	−0.148^***^	−0.321^***^	−0.153^***^	−0.102^***^	−0.134^***^	−0.130^***^	−0.133^***^
Economical adaptability	−0.020	−0.066^*^	−0.065	−0.121^***^		−0.030^*^		−0.046

*** *p < 0.001*,

** *p < 0.01, and*

* *p < 0.05*.

Secondly, we also performed Lasso regression to further confirm the key factors that can affect the mobile phone addiction. Results showed that the values of λ were 0.026, 0.001, 0.017, and 0.001, and λ+1se values were 0.171, 0.086, 0.267, and 0.121 at the minimum MSE through the training dataset when response data were from withdrawal, salience, social comfort, and mood change.

Finally, the retained variables were emotional adaptability, homesickness, and learning adaptability after bringing the best λ into the testing data, and they were accepted by *covTest* package. Results on Lasso regression in Table 4 show that emotional adaptability was negatively associated with withdrawal symptoms (β = −0.102, *p* < 0.001), salience (β = −0.134, *p* < 0.001), social comfort (β = −0.130, *p* < 0.001), and mood change (β = −0.133, *p* < 0.001). Homesickness adaptability was negatively associated with withdrawal symptoms (β = −0.005, *p* < 0.01) and salience (β = −0.065, *p* < 0.01), and learning adaptability was associated with salience (β = −0.159, *p* < 0.001).

## Discussion

### Main Findings

This study used traditional multiple regression to explore the associations between adaptability and nomophobia. Results showed that emotional adaptability, homesickness adaptability, and learning adaptability were significantly related to nomophobia. Emotional adaptability could negatively predict four dimensions of nomophobia (see Table 2). That is to say, individuals would exhibit more mobile phone uses when they have some emotional maladjustment (50). Results also showed that homesickness adaptability was negatively associated with fear of losing convenience, losing contact, and losing Internet connection (see Table 2). Results in this study were consistent with previous studies (51), which means that individuals, who have homesickness maladjustment, would have more demands for mobile phones, since mobile phones can help them to communicate with their families and friends anytime and anywhere (52) and relieve their homesickness maladjustment. In addition, we found that there was a negative association between learning adaptability and fear of losing convenience (see Table 2). Individuals would be more anxious if they could not use their mobile phone to access some learning resources (53, 54). After all, mobile phones had already been the most efficient tool to search for information.

Besides, this study mainly intended to explore which kinds of adaptability were closely related to nomophobia by using Lasso regression. Results showed that emotional adaptability was associated with fear of being unable to access information, losing convenience, and losing Internet connection after shrinking (see Table 3). In other words, not only would people be worried because of the dysfunction caused by missing out of mobile phone, but they also suffered from emotional maladjustment because of the absence of convenience provided by mobile phones (55). These viewpoints were verified again by the dataset on mobile phone addiction and adaptability. Those results showed that emotional adaptability was significantly associated with withdrawal symptoms, salience, social comfort, and mood change (see Table 4). Meanwhile, homesickness adaptability was negatively associated with withdrawal symptoms and salience (see Table 4).

Results also showed that the association between fear of losing contact and homesickness (β = −0.322, *p* < 0.001) was higher than with the emotional adaptability (β = −0.190, *p* < 0.01). That is to say, students' homesickness adaptability would lead to more demands for their phones, because mobile phones helped them to keep in touch with their families and friends (52, 56). We also found that the correlation between homesickness adaptability and fear of losing contact was higher than (*r* = −0.274, *p* < 0.01) other dimensions in nomophobia (*r* = −0.123, −0.152, −0.168, *p* < 0.001). It can also support the idea that homesickness adaptability predicted fear of losing contact.

This study provided the empirical evidence for the I-PACE model. The I-PACE model (27) supposed that there were many factors, such as biological factors, personality factors, and psychological factors, that affected mobile phone overuse. Our results supported this hypothesis and were consistent with the conclusion of previous studies (28). In sum, individuals' emotional cognition (adaptability in our study) resulted in some problematic behaviors related to smartphones (nomophobia and mobile phone addiction in this study) (43).

### Limitations and Future Directions

It should be noted that this study concluded that college students' academical adaptability, homesickness adaptability, and emotional adaptability could predict the level of nomophobia significantly. We just had found the factors that could affect nomophobia, but did not put forward effective measures to improve individuals' adaptability, because we just conducted a survey for the relationship between them. Therefore, we hope to solve this problem by an interventional study in the future. Besides, the relationship between adaptability and nomophobia was a cross-sectional study in this study. However, adaptability is a state variable to some extent. The results will be more persuasive if a longitudinal study is conducted. Therefore, a longitudinal design together with cross-lagged regression analysis is a good choice to verify the conclusions from this study.

### Implications

This study used Lasso regression to explore the relationship between individuals' adaptability and nomophobia. It has several implications. At first, it provided compelling evidence for the assumption of the I-PACE model by using empirical data, which confirmed that individuals' adaptability (psychological factors) could predict nomophobia significantly. Next, previous studies treated adaptability or nomophobia as a simple component (11, 13, 26), when the distinct dimensions of those two psychological traits were clarified. Furthermore, the specific relationships between different dimensions of adaptability and different dimensions of mobile phone overuse (mobile phone addiction and nomophobia) were examined. The key factors, such as emotional adaptability and homesickness adaptability, that affected nomophobia were discovered. Therefore, we further explained why individuals have various manifestations of nomophobia. Thirdly, this study also confirmed that the mobile phones bring both positive and negative effects on the participant. On the one hand, this study found that there were significant associations between mobile phone overuses with emotional adaptability and homesickness adaptability. On the other hand, for some college students, they could get in touch with their family through mobile phones when they suffer from homesickness maladjustment. It indicated that decreasing PUPM could be complex in practice.

## Conclusion

In conclusion, this study found that emotional adaptability was the key factor that can influence the level of fear of being unable to access information, losing convenience, and losing Internet connection. Homesickness adaptability was an important factor that can affect the fear of losing contact in nomophobia. That is to say, individuals' level of emotional adaptability and homesickness adaptability are important adaptability traits for predicting nomophobia. Individuals would exhibit the fear of being unable to get information, losing convenience, and losing Internet connection in nomophobia if they have some performance about emotional maladjustment. Additionally, they would exhibit the fear of losing contact in nomophobia if they have some performance about homesickness maladjustment.

## Data Availability Statement

The raw data supporting the conclusions of this article will be made available by the authors, without undue reservation.

## Ethics Statement

The studies involving human participants were reviewed and approved by XL2020-08. The patients/participants provided their written informed consent to participate in this study. We have informed the participants about the relevant content of the study before the test and have obtained the participants' consent. We also informed the participants that data will only be used for scientific research, and they have the right to automatically withdraw at any time.

## Author Contributions

JL: methodology and writing—reviewing and editing. SR: conceptualization, methodology, software, writing—original draft preparation, and writing—reviewing and editing. YL: data collection and investigation. TL: conceptualization, methodology, and writing—reviewing and editing. All authors contributed to the article and approved the submitted version.

## Funding

This study was funded by the National Natural Science Foundation of China (Grant Number 11801576), which provided help in our data collecting and also funded by the National Natural Science Foundation of China (Grant Number 31800945), which provided help in our data collecting and data analysis.

## Conflict of Interest

The authors declare that the research was conducted in the absence of any commercial or financial relationships that could be construed as a potential conflict of interest.

## Publisher's Note

All claims expressed in this article are solely those of the authors and do not necessarily represent those of their affiliated organizations, or those of the publisher, the editors and the reviewers. Any product that may be evaluated in this article, or claim that may be made by its manufacturer, is not guaranteed or endorsed by the publisher.
